# Butterfly eggs prime anti-herbivore defense in an annual but not perennial *Arabidopsis* species

**DOI:** 10.1007/s00425-024-04541-9

**Published:** 2024-10-03

**Authors:** Maryse A. P. Huve, Norbert Bittner, Reinhard Kunze, Monika Hilker, Mitja N. P. Remus-Emsermann, Luis R. Paniagua Voirol, Vivien Lortzing

**Affiliations:** 1 Microbiology, Institute of Biology, Dahlem Centre of Plant Sciences, Freie Universität Berlin, Königin-Luise-Str. 12-16, 14195 Berlin, Germany; 2 Applied Genetics, Institute of Biology, Dahlem Centre of Plant Sciences, Freie Universität Berlin, Albrecht-Thaer-Weg 6, 14195 Berlin, Germany; 3 Applied Zoology/Animal Ecology, Institute of Biology, Dahlem Centre of Plant Sciences, Freie Universität Berlin, Haderslebener Str. 9, 12163 Berlin, Germany

**Keywords:** Herbivory, Inducible plant defenses, Insect oviposition, *Pieris brassicae*

## Abstract

**Main conclusion:**

Unlike *Arabidopsis thaliana*, defenses of *Arabidopsis lyrata* against *Pieris brassicae* larval feeding are not primable by *P. brassicae* eggs. Thus, egg primability of plant anti-herbivore defenses is not phylogenetically conserved in the genus *Arabidopsis*.

**Abstract:**

While plant anti-herbivore defenses of the annual species *Arabidopsis thaliana* were shown to be primable by *Pieris brassicae* eggs, the primability of the phylogenetically closely related perennial *Arabidopsis lyrata* has not yet been investigated. Previous studies revealed that closely related wild Brassicaceae plant species, the annual *Brassica nigra* and the perennial *B. oleracea*, exhibit an egg-primable defense trait, even though they have different life spans. Here, we tested whether *P. brassicae* eggs prime anti-herbivore defenses of the perennial *A. lyrata*. We exposed *A. lyrata* to *P. brassicae* eggs and larval feeding and assessed their primability by (i) determining the biomass of *P.* *brassicae* larvae after feeding on plants with and without prior *P. brassicae* egg deposition and (ii) investigating the plant transcriptomic response after egg deposition and/or larval feeding. For comparison, these studies were also conducted with *A. thaliana.* Consistent with previous findings, *A. thaliana*’s response to prior *P. brassicae* egg deposition negatively affected conspecific larvae feeding upon *A. thaliana*. However, this was not observed in *A. lyrata*. *Arabidopsis thaliana* responded to *P. brassicae* eggs with strong transcriptional reprogramming, whereas *A. lyrata* responses to eggs were negligible. In response to larval feeding, *A. lyrata* exhibited a greater transcriptome change compared to *A. thaliana*. Among the strongly feeding-induced *A. lyrata* genes were those that are egg-primed in feeding-induced *A. thaliana*, i.e., *CAX3, PR1*, *PR5,* and *PDF1.4.* These results suggest that *A. lyrata* has evolved a robust feeding response that is independent from prior egg exposure.

**Supplementary Information:**

The online version contains supplementary material available at 10.1007/s00425-024-04541-9.

## Introduction

Plants employ various strategies to defend themselves against herbivorous insects, including constitutive and inducible anti-herbivore defenses. While constitutive defenses are always active, inducible anti-herbivore defenses are triggered only upon attack (War et al. 2012). Inducibility may save energy, allowing resources to be allocated to other vital processes such as growth and reproduction (Cipollini et al. 2003). Initiating inducible defenses incurs a time delay, resulting in a time lag between an insect’s attack and the plant’s corresponding defense response. During this interval, the plant remains susceptible to herbivore damage (Frost et al. 2008). However, this delay can be reduced if the plant detects early cues indicating imminent insect attacks. This process, known as “priming”, prepares the plant’s defense mechanisms to “anticipate” and counter threats. As a result of priming, the plant’s response to herbivory becomes quicker, more sensitive, and potentially stronger, as it “anticipates” these attacks (Hilker et al. 2016).

A wide range of environmental cues prime plant anti-herbivore defenses (Conrath et al. 2006; Pastor et al. 2013), for example, various volatile organic compounds, such as (i) feeding damage-induced plant volatiles (for example, Kost and Heil 2006; Dicke and Baldwin 2010), (ii) insect oviposition-induced plant volatiles (Pashalidou et al. 2020) or (iii) insect pheromones (Helms et al. 2017; Bittner et al. 2019). Besides airborne cues, direct interactions with herbivorous insects, such as footprints (Peiffer et al. 2009), chewing vibrations (Appel and Cocroft 2014), and feeding damage (Rasmann et al. 2012) can prime plant anti-herbivore defenses. In addition, insect egg depositions on leaves are highly reliable cues indicating impending herbivory by hatching larvae (Hilker and Fatouros 2015).

Egg deposition by herbivorous insects was shown to prime a wide range of plant species, including tree species (Beyaert et al. 2012; Austel et al. 2016), perennial shrubs (Pashalidou et al. 2015; Geuss et al. 2018) and herbaceous annual species (Geiselhardt et al. 2013; Bandoly et al. 2015, 2016; Pashalidou et al. 2015; Bonnet et al. 2017; Rondoni et al. 2018; Lortzing et al. 2019; Paniagua Voirol et al. 2020). For annual plant species, egg primability could be particularly beneficial because they produce seeds only once in their life cycle. As the life cycle of annual plants is very short, it has been hypothesized that the primability of plant defenses by responses to insect egg deposition allows annual plants to react faster to subsequent herbivore attacks. Mitigation of severe damage by priming a plant’s anti-herbivore defense by prior insect egg deposition may support recovery from damage and thus, seed set (Valsamakis et al. 2022). Anti-herbivore defense in perennial plants has also been investigated for primability by responses to insect egg deposition (Beyaert et al. 2012; Pashalidou et al. 2015, 2020; Austel et al. 2016; Geuss et al. 2018). Perennial plants might have long term benefits from priming their anti-herbivore defenses not only for the current season, but also the future one (Haukioja et al. 1985; Schott et al. 2023). The evolution of primable traits in annual and perennial plants might be driven by various factors, among them the plant’s “memory” abilities as well as environmental factors like the predictability of stress or the community in which the plant is living (Hilker et al. 2016). Specifically, endogenous properties of the organism such as the life span of a plant, as well as environmental factors (resource availability, predictability of the presence of intra- and inter-specific competitors and stressors), but also the metabolic and energy costs of a given stress response shape the plant’s ability to “remember” and may affect the evolution of primability. For example, mounting a constitutive defense against a given stress may become more cost-efficient than maintaining an induced state if the stress is a constant part of the environment. The benefits of being in a primed state must outweigh the costs of remembering and processing the initial priming exposure in order to favor evolution of the primability of this trait (Hilker et al. 2016).

Previous studies highlighted that egg deposition by the Large White butterfly (*Pieris brassicae*) primes various Brassicaceae species, leading to an impaired development of *P.* *brassicae* larvae. *Pieris brassicae* larvae feeding on prior egg-laden annual *Arabidopsis thaliana* consumed less leaf tissue, gained less biomass and suffered higher mortality in comparison to *P.* *brassicae* larvae feeding on egg-free plants (Geiselhardt et al. 2013; Valsamakis et al. 2022). This effect is elicited by a sticky, exocrine secretion produced by the butterfly female’s accessory reproductive gland, which attaches *P.* *brassicae* eggs to the leaf surface. The intensity of this effect acts in a dose-dependent manner, varying with the amount of secretion perceived by the plant (Paniagua Voirol et al. 2020), and it is mediated by the phytohormone salicylic acid (SA), involving the expression of *PR5* together with the accumulation of phenylpropanoid derivatives (Lortzing et al. 2019). Studies suggest that phosphatidylcholine-based compounds from the secretion of the accessory reproductive gland are likely the compounds eliciting egg-induced plant responses (Stahl et al. 2020; Lortzing et al. 2024). Furthermore, egg-primed responses are adjusted to the hatching time point of *P. brassicae* larvae. Thereby, the expression of SA-responsive genes *PR1* and *PR5*, as well as cation exchanger-coding *CAX3* and plant defensin-coding *PDF1.4* gradually increased during the egg incubation period (Valsamakis et al. 2020). Apart from *A. thaliana*, also the defenses of other annual Brassicaceae are primable by *P.* *brassicae* eggs: *Brassica nigra*, *Sinapis arvensis*, and *Moricandia moricandioides* (Pashalidou et al. 2013, 2015). So far, the primability of anti-herbivore defenses by *P. brassicae* eggs has only been described in one perennial Brassicaceae, *Brassica oleracea* (Pashalidou et al. 2015, 2020). This raises the question whether also other perennial Brassicaceae species are primable by butterfly eggs and whether their primability is comparable to annual Brassicaceae species.

The aim of this study was to investigate the egg primability of the perennial *Arabidopsis lyrata* (Al-Shehbaz and O’Kane 2002) and to compare it with the well-known egg primability of the phylogenetically closely related, annual species *A. thaliana* (Geiselhardt et al. 2013; Lortzing et al. 2019; Paniagua Voirol et al. 2020; Valsamakis et al. 2020, 2022). Both plant species share a high degree of genome sequence similarity (Nasrallah 2000), but have different life spans and grow in different environmental conditions (Al-Shehbaz and O’Kane 2002). *Arabidopsis thaliana* occurs as a winter annual on sandy soil, roadsides, rocky slopes, waste places, cultivated ground and meadows (Al-Shehbaz and O’Kane 2002), whereas *A. lyrata* is a drought-tolerant pioneer herb occurring in low-competition environments such as cliffs, calcareous ledges, rock crevices and sandy areas, e.g., with calcium-deficient serpentine soils (Al-Shehbaz and O’Kane 2002; Turner et al. 2010; Sletvold and Ågren 2012). Such environments with low plant density have been shown to negatively affect herbivory (Vergeer and Kunin 2011). Both species have basally pilose and apically glabrous stems, and trichomes on their leaves (Al-Shehbaz and O’Kane 2002). In both species, trichome density was shown to vary in response to herbivory and contributed to increase resistance to herbivore insects (Handley et al. 2005; Sletvold et al. 2010). Both *A. thaliana* and *A. lyrata* contain similar glucosinolates profiles (Clauss and Koch 2006). Glucosinolates are sulfur- and nitrogen-containing secondary plant metabolites, which are known to mainly target generalist herbivore insects. However, glucosinolates and their volatile derivatives are attractants, stimulants and act as recognition cues for adapted lepidopterans such as *P.* *brassicae* (Louda and Mole 1991). Based on these strong similarities of *A. thaliana* and *A. lyrata*, we asked whether the two plant species respond similarly to insect egg deposition in spite of their different life span and habitats.

A previous study showed that *P. brassicae* eggs induce an egg-killing trait that is phylogenetically conserved within species of the Brassiceae tribe including *Brassica* crops and close relatives (Griese et al. 2021). Furthermore, the anti-herbivore defenses of the closely related *B. nigra* and *B. oleracea* plant species are primable by *P. brassicae* eggs (Pashalidou et al. 2013, 2015, 2020), although they have different life spans. We hypothesized that the anti-herbivore defenses of *A. lyrata* are as primable by *P. brassicae* eggs as those of *A. thaliana*. Therefore, we compared the primability of *A. thaliana* and *A. lyrata* by exposure of both plant species to *P. brassicae* eggs and larval feeding. We (i) determined the biomass of larvae after feeding on plants with and without prior egg deposition and (ii) investigated the plant transcriptomic response after egg deposition and/or larval feeding. Unlike *A. thaliana*, defenses of *A. lyrata* against *P. brassicae* larval feeding are not primable by *P. brassicae* eggs. Instead, *A. lyrata* exhibited a greater transcriptome change in response to larval feeding compared to *A. thaliana*. These results suggest that *A. lyrata* has evolved a robust feeding response that is independent of prior egg deposition.

## Materials and methods

### Plant material and growth conditions

Seeds of *Arabidopsis thaliana* (Columbia-0) and *A. lyrata* ssp. *lyrata* (RonC) were sown on a 3:1 mixture of soil (Einheitserde classic): sand and stratified for 2 days at 4 °C. The plants grew in climate chambers under short day conditions (10 h/14 h light/dark cycle, 21 °C, 40% relative humidity, 100–120 µmol m^−2^ s^−1^ light intensity). After 1 week, the seedlings were transplanted in individual pots (7 cm × 7 cm × 8 cm, Soparco, Condé-sur-Huisne, France). The pots were placed in 42 cm × 58 cm trays (Albert Treppens & Co Samen GmbH, Berlin, Germany). Plants were watered once or twice per week. Seven-week-old *A. thaliana* and *A. lyrata* were used for the experiments. Additionally, the experiment displayed in Suppl. Fig. S1 was conducted with nine-week-old *A. lyrata* plants.

### *Pieris brassicae* rearing

*Pieris brassicae* was reared as described by Valsamakis et al. (2022). Briefly, larvae and adult butterflies were kept in flight cages (45 cm × 45 cm × 60 cm) under long day conditions (18 h/6 h light/dark cycle, 220 µmol m^−2^ s^−1^ light intensity, 23 °C and 70% relative humidity). The larvae were fed with Brussels sprouts plants (*Brassica oleracea* var. *gemmifera*) until pupation. The adult butterflies were fed with 15% aqueous honey solution. Before treating plants with eggs, mated female butterflies were not offered any plant for egg deposition.

### Plant treatments

#### Treatment with eggs

We treated *Arabidopsis* plants with eggs (E) by placing one butterfly by hand on a single fully developed, non-senescent leaf (leaf position 17–22), resulting in the deposition of a clutch containing 30–40 *P.* *brassicae* eggs. The eggs remained on the plant leaves for 6 days, which is the typical incubation time until hatching at 20 °C (David and Gardiner 1962). After 6 days under short day conditions (10 h/14 h light/dark cycle, 21 °C, 40% relative humidity, 100–120 µmol m^−2^ s^−1^ light intensity), we carefully removed the eggs shortly before they hatched, using tweezers and a fine brush. Untreated plants were used as control (C).

#### Treatment with larvae

To treat experimental plants with larval feeding (F), eggs were collected on backup Brussels sprouts plants. One day before hatching, eggs were collected and placed in Petri dishes until the larvae hatched (10 h/14 h light/dark cycle, 21 °C, 40% relative humidity, 100–120 µmol m^−2^ s^−1^ light intensity). Ten randomly selected neonate larvae were placed on a single, fully developed, non-senescent leaf (leaf position 17–22) of a previously egg-laden or an egg-free *A. thaliana and A. lyrata* plant, respectively, and enclosed in one clip cage per plant (2 cm in diameter, 1.7 cm high). If the plant had previously undergone egg deposition (E + F plants), the egg clutch was removed and replaced by ten neonate larvae, enclosed in a single clip cage per plant, which was placed directly at the oviposition site.

After 2 days feeding within the clip cage, larvae were removed with a fine brush from the plant for biomass determination (see “Determination of larval biomass”). Thereafter, they were placed back to their respective plants and were allowed to freely move and feed on the entire plant. Three days later, they were removed again for an additional biomass determination after five days of feeding (see below). To prevent larvae from escaping during the feeding process, the plants were enclosed in Plexiglas® cylinders (14.5 cm diameter, 15 cm high) with a gauze lid. After the second biomass determination, the larvae were frozen and discarded.

### Determination of larval biomass

Larval performance was assessed by determining the biomass of *P. brassicae* larvae per plant with a fine-scale balance (Ohaus® Analytical Plus balance Ohaus AP250D, Nänikon, Switzerland). We calculated the average larval biomass after a 2-day and 5-day feeding period on 7-week-old *A. thaliana* and *A. lyrata* plants. Additionally, the larval biomass was determined after a 2-day and 5-day feeding period on 9-week-old, egg-free and previously egg-laden *A. lyrata* plants.

### Sampling of leaf tissue

For transcript analyses, the experiments were designed in 2 × 2 factorial setup. Plants were exposed to eggs (E), to larval feeding (F), or to both eggs and feeding (E + F). Untreated plants were used as control (C) (see “Plant treatments”). After 2 days of feeding on 7-week-old plants with or without eggs, we harvested leaf material for transcriptome analyses. Treated leaves were flash frozen in liquid nitrogen.

### RNA extraction

We extracted total RNA as described by Oñate-Sánchez and Vicente-Carbajosa (2008) and removed residual genomic DNA with the TURBO DNA free™ kit (ThermoFisher Scientific, Waltham, MA, USA) following the manufacturers recommendations. The RNA quantity and quality was inspected on a 1.2% agarose gel and with a Thermo Scientific™ Multiskan™ GO Microplate Spectrophotometer. RNA integrity (RIN between 6.6 and 8.8) was estimated using the Bioanalyzer 2100 (Agilent Technologies, Santa Clara, CA, USA) before samples were sequenced (Macrogen Europe, Amsterdam, The Netherlands).

### RNA sequencing and analysis of differentially expressed genes

The lllumina TruSeq Stranded Poly-A selected RNA Sample library kit for plants was used to prepare samples for sequencing. Paired end sequencing (2 × 150 bp) was conducted using the NovaSeq6000 platform (Illumina, San Diego, CA, USA). All samples produced between 32.5 and 36.6 million reads. For adapter clipping and trimming, Trimmomatic was used (version 0.39) (Bolger et al. 2014). Sequences shorter than 50 bp were excluded from further analysis. The sequence quality was inspected with FastQC and MultiQC before and after adapter clipping and trimming (Ewels et al. 2016; Wingett and Andrews 2018). Ribosomal sequences were filtered with SortmeRNA (version 2.1) (Kopylova et al. 2012). To map reads against the plant genomes, the genomes and their annotation from *A. thaliana* and *A. lyrata* were obtained from Ensembl Plants (Howe et al. 2020) (version TAIR10, release 44) and from Rawat et al. (2015). Reads were counted with kallisto (version 0.46.0) (Bray et al. 2016), and resulting count files were converted to the DESeq2 package data format with the tximeta package (Love et al. 2020) (Bioconductor version 3.9) (Soneson et al. 2015) in R (R Core Team 2022). All genes with a read count > 1 were considered for analysis of differential expression. Differentially expressed genes (DEGs) were defined to have a *P* value ≤ 0.05 after *fdr* correction for multiple testing.

For further downstream analysis of gene functions, we annotated *A. lyrata* genes to their *A. thaliana* orthologs by employing supplemental datasets of the latest *A. lyrata* annotation (Suppl. Data S1) (Rawat et al. 2015). The biological functions of DEGs were examined via enrichment analyses of Kyoto Encyclopedia of Genes and Genomes (KEGG) pathway and gene ontology (GO) terms with DAVID 6.8 (Sherman et al. 2022). The transcriptome data were further explored using R (R Core Team 2022) and Venn diagrams [package “eulerr” (Larsson 2024)], hierarchical clustering analyses, heatmapping using Euclidean distances, and Complete-linkage clustering [packages “ComplexHeatmap” (Gu et al. 2016), “dentextend” (Galili 2015)].

### cDNA synthesis and quantitative real-time PCR

First strand cDNA was synthesized from 2 µg RNA with the smART Reverse Transcriptase kit (Roboklon GmbH, Berlin, Germany) and oligo-dT18 following the manufacturer’s protocol. Quantitative real-time PCRs were conducted in a total of 10 µl using Blue S’Green qPCR 2 × Mix (Biozym Scientific GmbH, Hessisch Oldendorf, Germany). As reference genes, for *A. thaliana ACT2* (AT3G18780), *GADPH* (AT1G13440) and *TUB6* (AT5G12250), and for *A. lyrata PS2SUB, PYRT* and *PPP2R1′3* were used*.* All primers are listed in Suppl. Table S1. Relative expression of each gene was calculated with the ∆∆CT method (Livak and Schmittgen 2001).

### Statistics

Statistical evaluation and visualizing were performed with R (R Core Team 2022). The following packages were used: car (Fox and Weisberg 2019), cowplot (Wilke 2022), ggplot2 (Wickham 2016), ggpubr (Kassambara 2023), psych (Revelle 2024), reshape2 (Wickham 2007), Rmisc (Hope 2022), tidyverse version 1.3.0 (Wickham et al. 2019), viridis (Garnier et al. 2024).

Data distribution was evaluated with Shapiro–Wilk test and Q-Q-plot. Homogeneity of data variances was assessed using Levene’s test. For larval biomass data, we applied multiple Student’s *t*-test with *fdr* correction post hoc, because data were normally distributed and had homogenous variances. For qPCR data, we applied 2 × 2 factorial ANOVA with Tukey post hoc test based on normal distribution of data with homogenous variances.

## Results

### The plant’s response to *Pieris brassicae* eggs negatively affects larval growth on *Arabidopsis thaliana* but not on *Arabidopsis lyrata*

**Fig. 1:**
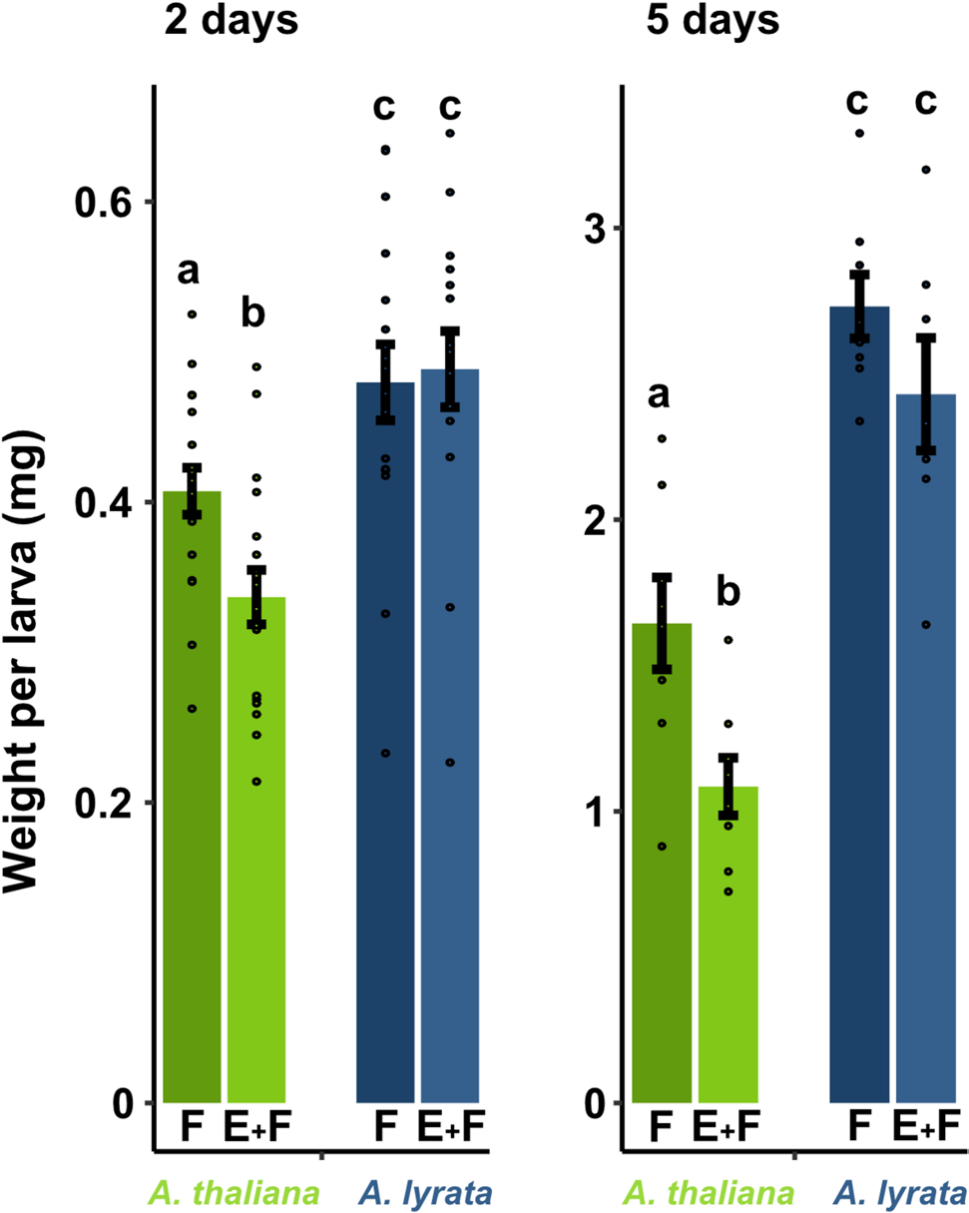
Impact of the plant’s responses to *Pieris brassicae* eggs on biomass of conspecific larvae feeding on *Arabidopsis thaliana* (green) and *A. lyrata* (blue) plants. Biomass in mg (means ± SE; after 2 days feeding *n* = 16–18, after 5 days feeding *n* = 7–8) of larvae after 2 or 5 days feeding on previously egg-laden (E + F) and egg-free (F), 7-week-old plants. Dots represent the data points. Different letters above the bars indicate significant differences between treatments and plant species (*P* < 0.05, multiple Student’s *t*-test with *fdr* correction). Statistical details are provided in Suppl. Table S2

We assessed larval biomass after 2 and 5 days of feeding on 7-week-old *A.* *thaliana* and *A. lyrata*, with or without prior exposure to *P. brassicae* eggs (Fig. 1). Larvae feeding on previously egg-laden *A. thaliana* plants gained significantly less biomass compared to those feeding on egg-free plants, regardless of the feeding duration. By contrast, prior egg deposition on *A. lyrata* did not affect larval biomass. This lack of an egg-priming effect on larval biomass was observed in both 7-week-old (Fig. 1) and 9-week-old *A. lyrata* plants (Suppl. Fig. S1). Moreover, larvae gained significantly more biomass when feeding on *A.* *lyrata* compared to *A. thaliana* (Fig. 1).

### *Arabidopsis lyrata* responds to *Pieris brassicae* eggs with a weaker transcriptional reprogramming than *Arabidopsis thaliana*

**Fig. 2:**
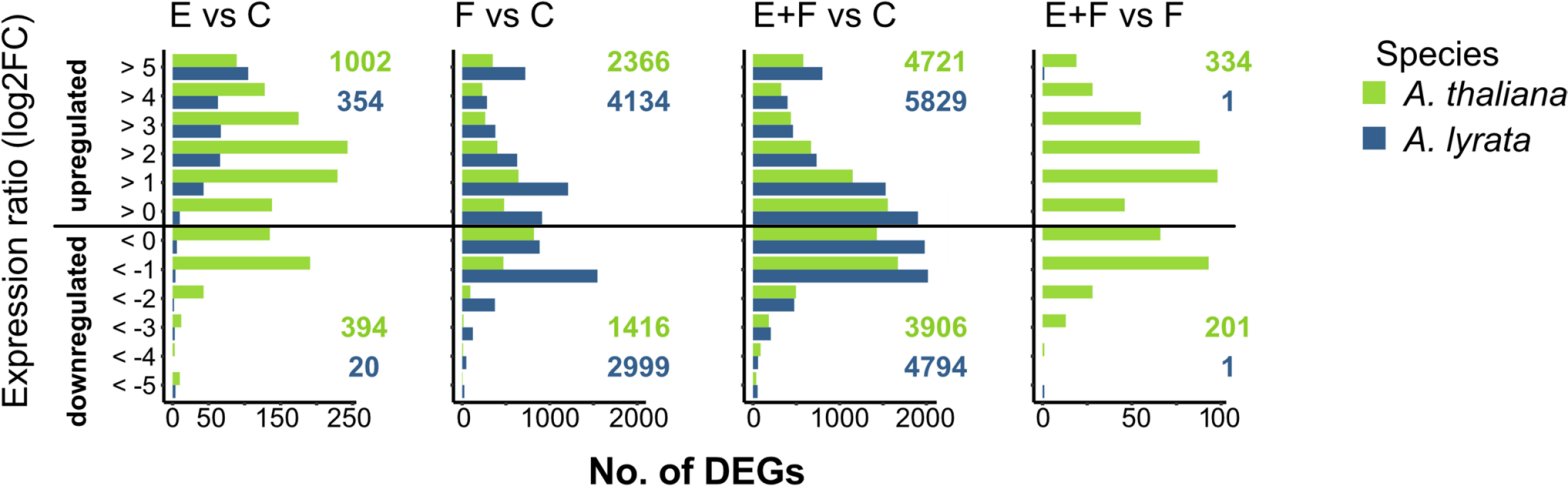
Number of differentially expressed genes (DEGs) in *A. thaliana* (green) and *A. lyrata* (blue) in response to *Pieris brassicae* eggs (E), larval feeding (F) or both, eggs followed by larval feeding (E + F) for the following treatment comparisons: E versus C, F versus C, E + F versus C and E + F versus F. Control plants (C) were left untreated. Expression ratio is represented as a log_2_ fold change (log_2_FC; *n* = 5). Gene counts from kallisto were normalized with DESeq2 median-of-ratio method. The fold change was calculated as follow: fold change (FC) = log_2_$\left(\frac{normalized\; counts\; group\; 1}{normalized \;counts\; group\; 2}\right)$

**Fig. 3:**
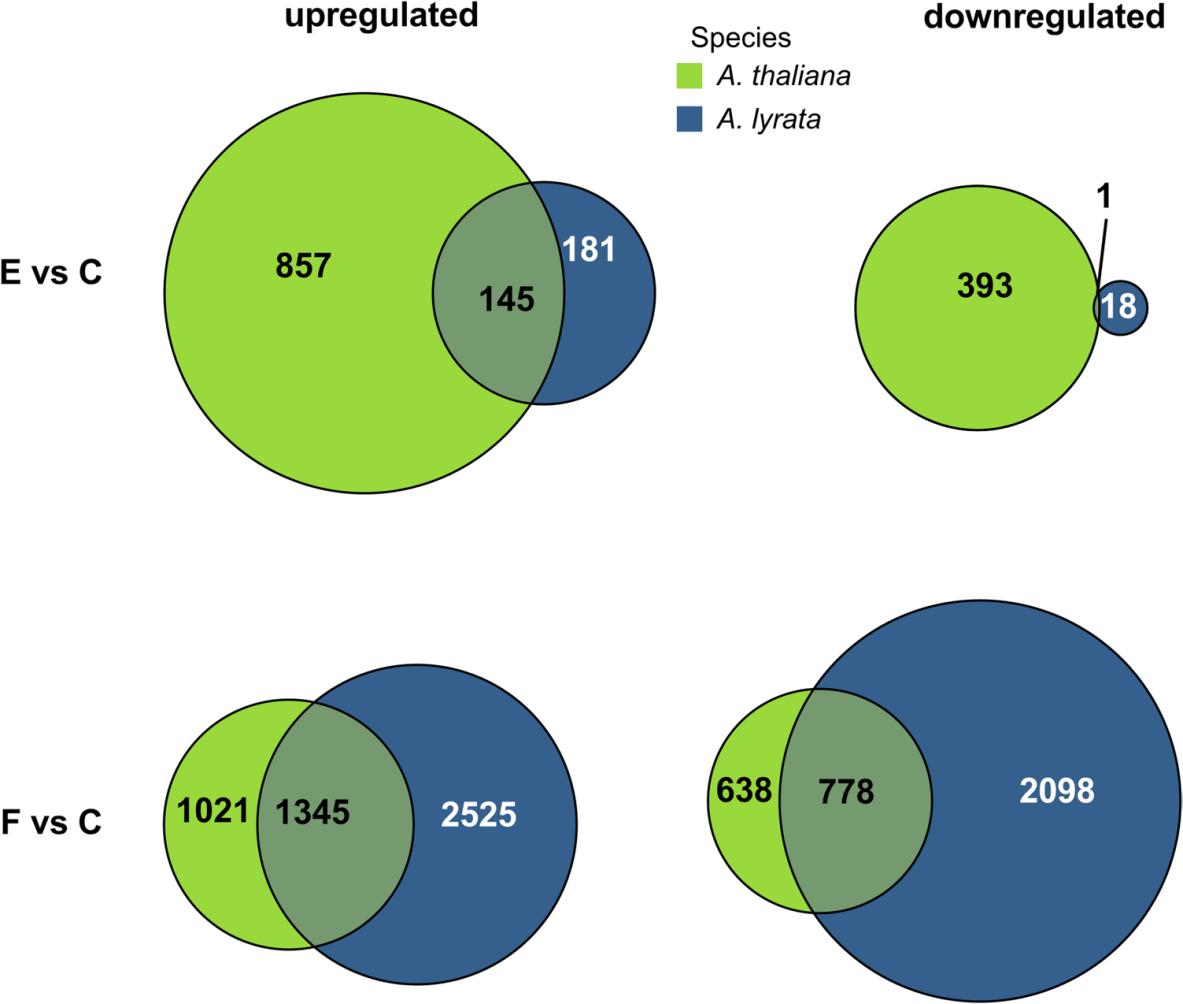
Common and unique differentially expressed genes in *Arabidopsis thaliana* and *A. lyrata* (*n* = 5) in response to *Pieris brassicae* eggs (E vs C) or larval feeding (F vs C). Circular areas are drawn to scale relative to the number of DEGs. *Arabidopsis lyrata* genes without *A. thaliana* orthologs are not shown in this figure but are listed in Suppl. Data S1

We investigated the effect of *P. brassicae* eggs on the transcriptomes of 7-week-old *A. lyrata* and *A. thaliana* plants using RNA-seq. In response to *P.* *brassicae* eggs, *A. lyrata* showed fewer DEGs than *A. thaliana* (Fig. 2). *Arabidopsis thaliana* responded to *P. brassicae* eggs with strong transcriptional reprogramming. Overall, 1396 *A. thaliana* genes were differentially expressed (1002 up-, 394 downregulated genes) (Fig. 2, Suppl. Data S1). This represents 5% of the total number of protein coding genes of *A. thaliana* (Cheng et al. 2017). The transcriptomic response of *A. lyrata* to *P.* *brassicae* eggs was much weaker compared to *A. thaliana*, with *A. lyrata* exhibiting 326 upregulated genes and 19 downregulated genes in response to the eggs (Fig. 3, E vs C, Suppl. Data S1), which represents 1% of the total number of protein coding genes in *A. lyrata* (Hu et al. 2011).

### Upregulation of genes in response to oviposition

**Fig. 4:**
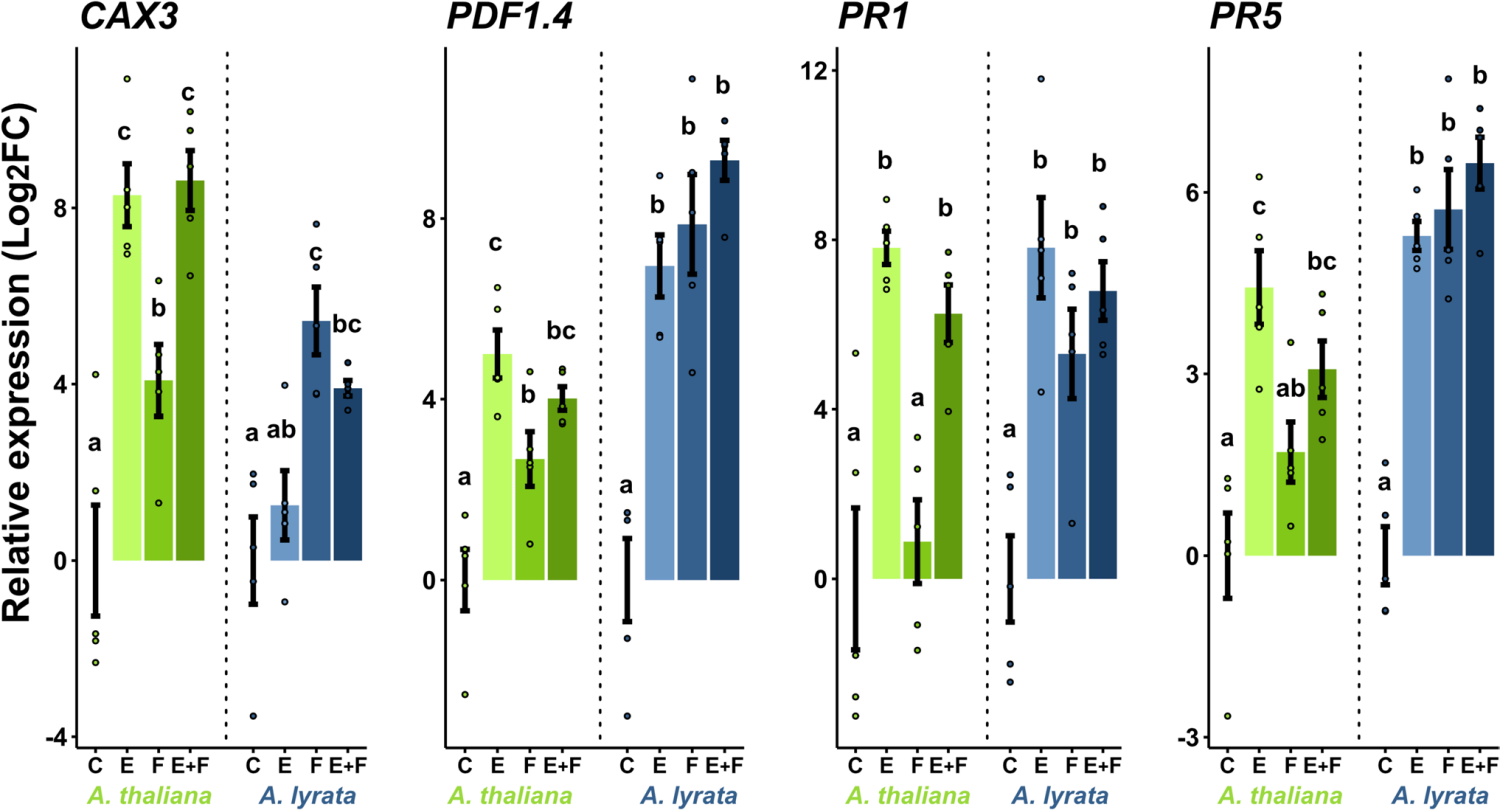
Relative expression of the priming-responsive genes *CAX3*, *PDF1.4*, *PR1* and *PR5* in *Arabidopsis thaliana* (green) and *A. lyrata* (blue). The plants were exposed to *Pieris brassicae* eggs (E), larval feeding (F) or eggs with subsequent larval feeding (E + F) or were left untreated (C). Bars indicate mean relative expression (Log_2_FC ± SE, *n* = 4–5), dots represent individual data points. Different letters above the bars indicate significant differences between treatments (*P* < 0.05, 2 × 2 ANOVA with Tukey test post hoc). Detailed statistics are provided in Suppl. Table S3

Nearly half (44%) of the orthologous egg-inducible genes in *A. lyrata* were also found to be inducible in *A. thaliana* (Fig. 3, E vs C, Suppl. Data S4). These egg-inducible genes in both *Arabidopsis* species include well-known insect egg-responsive markers like *PR5* and *PDF1.4*, which showed similarly strong egg inducibility in both plant species (Suppl. Data S4). In an independent experiment, qPCR analysis confirmed that both plant species exhibited similarly strong expression of *PDF1.4, PR1* and *PR5* in response to eggs, while the expression of *CAX3* was considerably stronger in *A. thaliana* than in *A. lyrata* (Fig. 4).

**Fig. 5:**
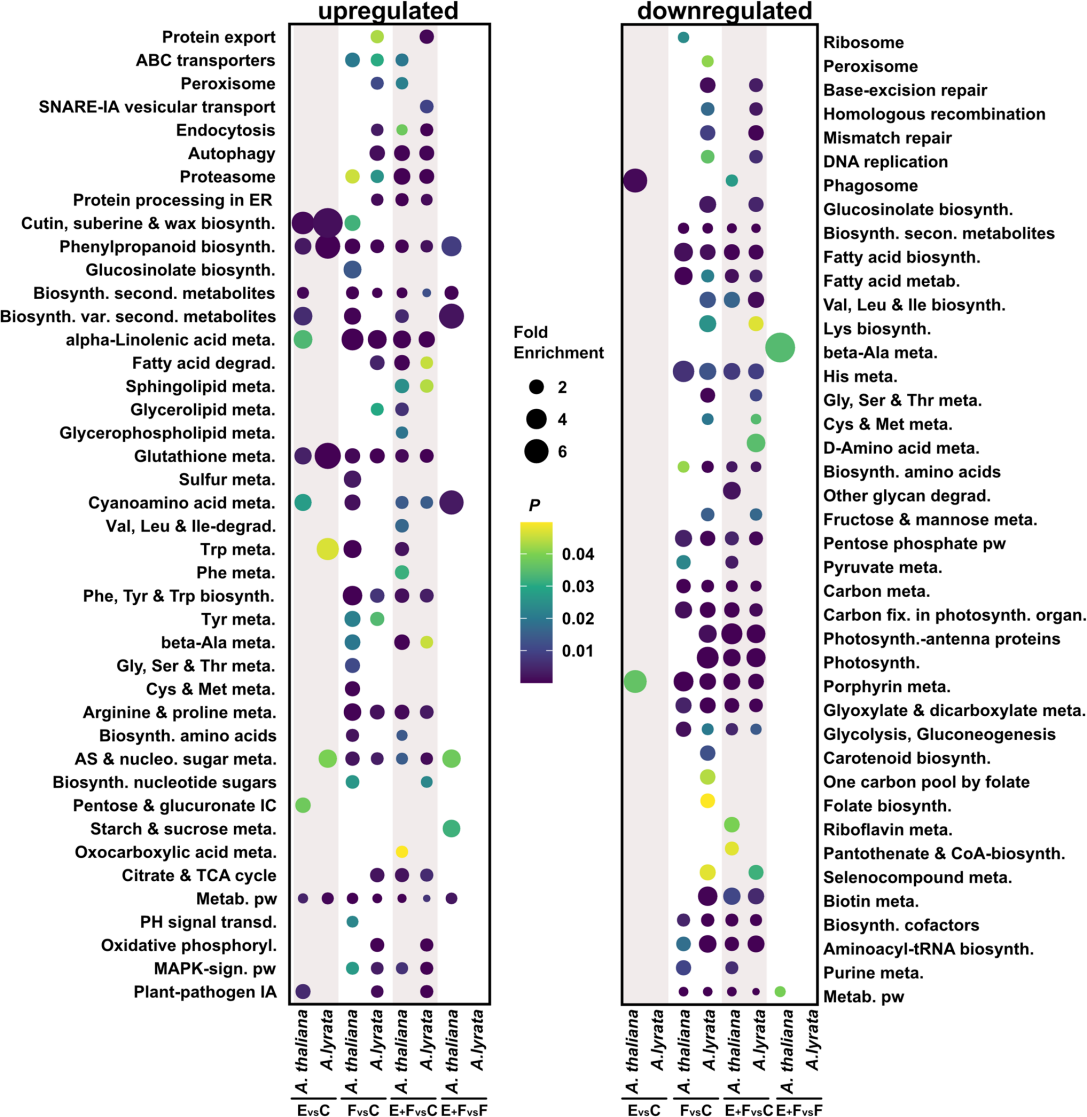
KEGG pathway enrichment analysis of the transcriptional responses of seven-week-old *Arabidopsis thaliana* and *A. lyrata* to *Pieris brassicae* eggs (E), larval feeding (F) or both, eggs followed by larval feeding (E + F). Control plants were left untreated (C). Enrichments in KEGG pathways were made for the following treatment comparisons: E versus C, F versus C, E + F versus C and E + F versus F. The circle size represents fold enrichment of genes in the KEGG pathway, the color indicates the *P* value (*n* = 5). *Ala* alanine, *AS* amino sugar, *biosynth.* biosynthesis, *Cys* cysteine, *degrad.* degradation, *ER* endoplasmic reticulum, *fix* fixation, *Gly* glycine, *His* histidine, *IA* interaction, *IC* interconversions, *Ile* isoleucine, *Leu* leucine, *Lys* lysine, *Met* methionine, *meta.* metabolism, *metab.* metabolic, *nucleo* nucleotide, *PH* plant hormone, *Phe* phenylalanine, *phosphoryl.* phosphorylation, *pw* pathway, *second.* secondary, *Ser* serine, *sign.* signaling, *TCA* tricarboxylic acid, *Thr* threonine, *transd.* transduction, *Trp* tryptophan, *Tyr* tyrosine, *Val* valine, *var.* various

The upregulated genes in egg-laden *A. lyrata* were enriched in less KEGG pathway and GO terms than in *A. thaliana* [KEGG pathway term enrichment: 6 terms for *A. lyrata* and 10 terms for *A. thaliana* (Fig. 5, Suppl. Data S2); GO term enrichment: 63 terms for *A. lyrata* and 98 terms for *A. thaliana* (Suppl. Data S3)].

Similar to *A. thaliana*, upregulated genes in *A. lyrata* in response to *P. brassicae* eggs were enriched in the KEGG pathway term phenylpropanoid biosynthesis [e.g., *PEROXIDASE 52* (*PRX52*)] as well as in GO terms related to SAR (e.g., *PR5*), SA-mediated signaling, e.g., *WRKY DNA-BINDING PROTEIN*s including *WRKY18, WRKY46, WRKY60*), JA-mediated signaling [e.g., *JASMONIC ACID OXIDASE* 3 (*JOX3*)], and oxidative stress [e.g., *SENESCENCE ASSOCIATED GENE 14* (*SAG14*)] (Fig. 5, Suppl. Data S2 and S3).

However, in *A. lyrata* fewer genes were enriched in these GO terms than in *A. thaliana*. For example, specific genes like *DIHYDROFLAVONOL 4-REDUCTASE* (*TT3*), *ANTHOCYANIDIN SYNTHASE* (*ANS*), *FLAVONOL SYNTHASE* (*FLS*) *5* (phenylpropanoid biosynthesis), *ALLENE OXIDE CYCLASE* (*AOC*) *1*, *AOC3*, and *OXOPHYTODIENOATE-REDUCTASE 3* (*OPR3*)*, JASMONATE INSENSITIVE 1* (*MYC2*) (JA pathway), *AVRPPHB SUSCEPTIBLE 3* (*PBS3*)*, ENHANCED DISEASE SUSCEPTIBILITY 5* (*EDS5*) (SA pathway)*,* were upregulated only in previously egg-laden *A. thaliana* (Suppl. Data S3 and S4). Furthermore, *A. thaliana* responded to eggs with the upregulation of genes enriched in GO terms associated to calcium-mediated signaling and calcium ion homeostasis, e.g., 8 *CALMODULIN-LIKE* (*CML*) genes, *CALMODULIN 3* (*CAM3*) and *CALMODULIN 8* (*CAM8*), 3 *CALCIUM-DEPENDENT PROTEIN KINASE* (*CPK*) genes and *CAX3* (Suppl. Data S3 and S4). This response to *P. brassicae* eggs is absent in *A. lyrata* (Suppl. Data S3). This is also reflected by our qPCR data showing that *CAX3*, encoding for a Ca^2+^/H^+^ exchanger (Manohar et al. 2011), is upregulated in egg-laden *A. thaliana*, but not in egg-laden *A. lyrata* (Fig. 4).

### Downregulation of genes in response to oviposition

*Arabidopsis lyrata* downregulated only 20 genes in response to *P. brassicae* eggs (less than 0.5% of all downregulated genes in *A. lyrata*), whereas *A. thaliana* downregulated 394 genes in response to *P.* *brassicae* eggs (43% of all downregulated genes in *A. thaliana*) (Fig. 2).

Due to the limited number of downregulated genes in *A. lyrata* in response to eggs, the KEGG and GO term analyses lacked sufficient counts for meaningful interpretation. Genes downregulated in egg-laden *A. thaliana* were enriched in KEGG pathways and GO terms linked to chlorophyll biosynthesis [e.g., porphyrin metabolism, *PROTOCHLOROPHYLLIDE OXIDOREDUCTASE A* (*PORA*)], cell cycle and cell division [e.g., *CELL DIVISION CYCLE 20.1* (*CDC20.1*)] (Fig. 5, Suppl. Data S2 and S3).

Only one gene, *GDPDL4*, was downregulated in both *A. lyrata* and *A. thaliana* in response to eggs (Fig. 3, E vs C, Suppl. Data S4). *GDPDL4* encodes a protein with glycerophosphoryl diester phosphodiesterase-like activity, which plays a role in cellulose accumulation and pectin linking (Hayashi et al. 2008).

### *Arabidopsis lyrata*’s transcriptome response to *Pieris brassicae* larval feeding is stronger than that of *A. thaliana*

We determined the impact of *P. brassicae* larval feeding on the transcriptomes of 7-week-old *A.* *lyrata* and *A. thaliana* plants. Both *A. lyrata* and *A. thaliana* exhibited a strong transcriptional reprogramming in response to larval feeding (Fig. 2, Suppl. Data S1). The transcriptional reprogramming in *A. thaliana* was stronger, when plants were previously exposed to the eggs (Fig. 2, E + F vs. F). This could not be observed in *A. lyrata*. However, upon feeding damage alone (F vs C), 1.6 times more genes were up- or down-regulated in *A.* *lyrata* than in *A. thaliana*, with 7142 protein coding genes showing a response in *A. lyrata* (4143 up- and 2999 down-regulated genes) compared to 3782 genes in *A. thaliana* (2366 up- and 1416 down-regulated genes) (Fig. 2, Suppl. Data S1).

When considering the genes with orthologs in both species, more than 50% that were regulated in *A. thaliana* by larval feeding were also regulated in *A. lyrata* (57% for upregulated, 55% for downregulated genes). However, *A. lyrata*’s transcriptional reprogramming was stronger in response to larval feeding than that of *A. thaliana* (Fig. 3, F vs C, Suppl. Data S4). The qPCR-analyses of egg-priming responsive genes also indicated that *CAX3*, *PDF1.4*, *PR1* and *PR5* were much stronger induced in *A. lyrata* in response to larval feeding than in *A. thaliana* compared to the untreated control plants (Fig. 4).

### Upregulation of genes in response to larval feeding

Although larval feeding induced more of the orthologous genes in *A. lyrata* than in *A. thaliana*, the KEGG pathway and GO term analysis resulted in similar numbers of enriched terms for both plant species [KEGG pathway term enrichment: 22 terms for *A. lyrata* and 24 terms for *A. thaliana* (Fig. 5 and Suppl. Data S2); GO term enrichment: 166 terms for both plant species (Suppl. Data S3)].

Both in *A. lyrata* and in *A. thaliana*, upregulated genes responding to larval feeding were significantly enriched in KEGG pathway terms associated with phenylpropanoid biosynthesis, biosynthesis of secondary metabolites, glutathione metabolism and alpha-linolenic acid metabolism (Fig. 5). Furthermore, commonly upregulated genes were enriched in GO terms associated to oxidative stress, ABA [*RESPONSE TO ABA AND SALT 1* (*RAS1*), *NDR1/HIN1-LIKE 6* (*NHL6*)], JA [*ACYL-COA OXIDASE 1* (*ACX1*), *LIPOXYGENASE 2* and *3* (*LOX2, LOX3*)] and SA synthesis and signaling [*ENHANCED DISEASE SUSCEPTIBILITY* 5 (*EDS5*), *12-OXOPHYTODIENOATE REDUCTASE 1* (*OPR1*)], and to the synthesis of flavonoids [*TRANSPARENT TESTA 8* (*TT8*)], including anthocyanins [*ANTHOCYANIDIN SYNTHASE* (*ANS*), *PRODUCTION OF ANTHOCYANIN PIGMENT* (*PAP1*)] (Suppl. Data S3 and S4).

Additionally, in *A. lyrata*, the feeding-induced genes were significantly enriched in a KEGG pathway term involved in plant-pathogen interactions (e.g., *CPK32*, *CPK1*) (Fig. 5, Suppl. Data S2). In comparison to *A. thaliana*, more feeding-induced *A. lyrata* genes were enriched in GO terms associated to unfolded protein response (e.g., endoplasmic reticulum unfolded protein response and response to endoplasmic reticulum stress) and to SA signaling (e.g., regulation of SA biosynthetic process and regulation of SA mediated signaling pathway) (Suppl. Data S3).

In *A. thaliana,* the feeding-induced genes were additionally significantly enriched in KEGG pathway terms related to glucosinolate biosynthesis [e.g., *SULFOTRANSFERASE 16 and 17* (*SOT16*, *SOT17*)], biosynthesis of various secondary metabolites and plant hormone signal transduction [*JASMONATE ZIM-DOMAIN PROTEIN 1* (*JAZ1*)] (Fig. 5, Suppl. Data S2).

### Downregulation of genes in response to larval feeding

In both plant species, exposure to larval feeding downregulated genes associated with the KEGG pathway terms biosynthesis of secondary metabolites, fatty acid biosynthesis and metabolism, carbon metabolism, fixation in photosynthetic organisms, and porphyrin metabolism (Fig. 5). Additionally, the GO term analysis showed that in both plant species, photosynthesis-related genes were downregulated in response to larval feeding (Suppl. Data S3).

Overall, the downregulated genes in feeding-damaged *A. lyrata* were enriched in more KEGG pathway and GO terms than in *A. thaliana* [KEGG pathway term enrichment: 32 terms for *A. lyrata* and 17 terms for *A. thaliana* (Fig. 5, Suppl. Data S2); GO term enrichment: 121 terms for *A. lyrata* and 86 terms for *A. thaliana* (Suppl. Data S3)].

In feeding-damaged *A. lyrata,* overall more downregulated genes were significantly enriched in KEGG pathway and GO terms that are associated to photosynthesis and pigment synthesis, e.g., KEGG pathways: photosynthesis-antenna proteins, photosynthesis [(*PHOTOSYSTEM II REACTION CENTER PSB28 PROTEIN* (*PSB28*), *PHOTOSYSTEM I SUBUNIT D-2* (*PSAD-2*)], chlorophyll synthesis (*CHLD* encoding a subunit of the magnesium chelatase), carotenoid biosynthesis, and glucosinolate biosynthesis [e.g., *ISOPROPYL MALATE ISOMERASE LARGE SUBUNIT 1* (*IIL1*), *SOT17*] (Fig. 5, Suppl. Data S2).

### Responses to eggs exert negligible effects on larval feeding-damaged *Arabidopsis lyrata*

Finally, we compared the impact of *P. brassicae* eggs and *P. brassicae* larval feeding on the transcriptomes of 7-week-old *A. thaliana* and *A. lyrata* with RNA-seq.

**Fig. 6:**
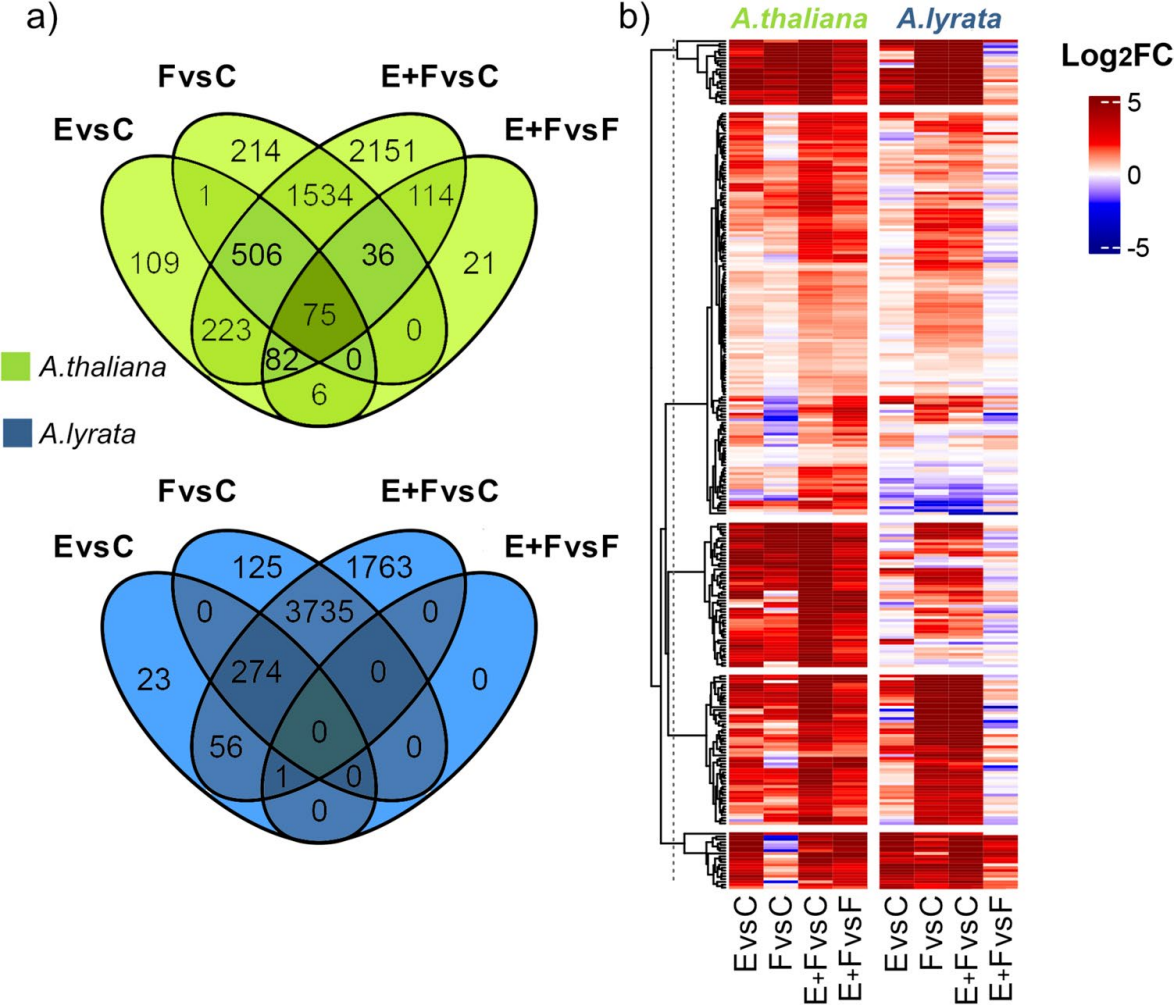
Impact of *Pieris brassicae* eggs and larval feeding to the primed transcriptome of *Arabidopsis thaliana* or *A. lyrata*. **a** Venn diagrams indicate the number of upregulated genes in *A. thaliana* (green) and *A. lyrata* (blue) that are commonly or uniquely regulated in response to *P. brassicae* eggs (E), larval feeding (F) or eggs and larval feeding (E + F). C represents transcriptomes of untreated plants. **b** Hierarchically clustered heatmap of up- or down-regulated *A. thaliana* and *A. lyrata* genes for the following treatment comparisons: E versus C, F versus C, E + F versus C and E + F versus F (log_2_FC; *n* = 5). Red colors indicate upregulation, blue colors downregulation of genes

In *A. thaliana*, 58% of egg-inducible genes (E vs C) were also induced in response to larval feeding (F vs C) (582 genes) (Fig. 6a). In *A.* *lyrata,* 77% of egg-responsive genes were also induced by larval feeding (274 commonly upregulated genes from 354 in total; Fig. 6a). However, compared to *A. thaliana*, *A. lyrata* upregulated less than 30% of the genes in response to eggs (Fig. 3, E vs C). From in total 5977 upregulated genes throughout all treatments, only 354 (less than 6%) were upregulated in response to eggs in *A. lyrata* (Fig. 6a).

When comparing the upregulated DEGs in E + F plants versus those in F plants in *A. lyrata,* we identified one cluster of genes that tended to be upregulated (Fig. 6b). However, only one gene (AT2G45340, encoding a leucine-rich repeat protein kinase family protein) was significantly upregulated in the E + F plants compared to F plants (E + F vs F, Fig. 6a, Suppl. Data S1 and S5).

By contrast, *A. thaliana* upregulated 334 genes in E + F plants compared to only feeding-damaged F plants (Fig. 6a, Suppl. Data S1). Among those are *CAX3* and *PR1* (Fig. 4), which were identified as egg-primed genes in previous studies (Little et al. 2007; Bruessow et al. 2010; Lortzing et al. 2019; Valsamakis et al. 2022). These 334 upregulated genes play a role, for example, in biosynthesis of phenylpropanoids [e.g., several genes coding for peroxidases (*PER23*, *PER50*, *PER54*, *PER58*), *4CLL7* that encodes 4-coumarate-CoA ligase-like 7] and in immune responses (e.g., *WRKY41*, *WRKY47*) (Fig. 5, Suppl. Data S2 and S3).

After egg deposition, both with and without subsequent larval feeding (E and E + F treatments), *A. thaliana* showed a stronger response than *A. lyrata* by upregulating genes related to “response to oxidative stress” (3.88- and 2.38-fold vs. 3.43- and 1.93-fold enrichments in comparison to their respective control plants) and “cellular response to oxidative stress” (3.4- and 1.85-fold enrichment, while *A. lyrata* did not show this response). Both species also upregulated genes associated with “response to oxidative stress” after feeding damage alone (F treatment).

Similar patterns were detected for downregulated genes when comparing E + F vs F plants (Suppl. Fig. S2a). Whereas in *A.* *lyrata* only one gene was downregulated in E + F plants when compared to F plants (AT4G33550, encoding a bifunctional inhibitor/lipid-transfer protein/seed storage 2S albumin superfamily protein) (Suppl. Data S1), *A. thaliana* E + F plants downregulated 201 genes when compared to F plants (Suppl. Fig. S2a). These 201 genes were involved, for example, in chlorophyll biosynthesis [e.g*., UROPORPHYRINOGEN III SYNTHASE* (*DUF3*)] and in photosynthesis [e.g., *PHOTOSYSTEM II SUBUNIT T* (*PSBTN*)] (Suppl. Data S3).

The small transcriptomic differences between egg-laden and feeding-damaged (E + F) *A. lyrata* plants and plants that were only fed upon (F), compared to the response of equally treated *A. thaliana* indicates the absence of an egg-mediated priming response in *A. lyrata* (Fig. 6b, Suppl. Fig. S2b).

## Discussion

Our study showed that defenses of the perennial Brassicaceae *A. lyrata* against larvae of *P. brassicae* are not primable by prior egg deposition. This is in contrast to previously investigated annual Brassicaceae and the perennial *B. oleracea*, which shows primed defenses against feeding after egg deposition (Pashalidou et al. 2013, 2015, 2020). Here, we compared the egg-primability of the anti-herbivore defense of *A. lyrata* with the closely related annual *A. thaliana*.

Previously, it was shown that *P.* *brassicae* larvae gain less biomass on previously egg-laden *A. thaliana* plants compared to larvae on egg-free plants (Geiselhardt et al. 2013; Lortzing et al. 2019; Paniagua Voirol et al. 2020; Valsamakis et al. 2020, 2022). By contrast, we found no priming effect in *A. lyrata*, i.e., the larvae performed equally well on previously egg-laden and egg-free plants (Fig. 1). Additionally, larvae feeding on *A. lyrata* gained significantly more biomass compared to larvae feeding on *A. thaliana*, possibly due to different feeding intensities of the larvae based on different nutrient contents or secondary metabolite profiles of the two plant species (Hwang et al. 2008; Pashalidou et al. 2015). The absence of the priming effect in *A. lyrata* is also reflected by its transcriptomic response to *P. brassicae* eggs with subsequent larval feeding. Our RNA-seq analysis revealed that only one gene was induced in egg deposited, feeding-damaged *A. lyrata* compared to feeding-damaged plants (Fig. 2 and Fig. 6a, Suppl. Data S1). This gene was AT2G45340 and is encoding for a leucine-rich repeat protein kinase family protein. In contrast to *A. lyrata*, prior egg deposition on feeding-damaged *A. thaliana* resulted in stronger induction of 334 genes compared to feeding-damaged plants without prior egg deposition (Figs. 2 and 6a), including typically egg priming-responsive genes like *PR1*, *PR5* and *CAX3* and several *WRKY* transcription factor genes (Fig. 4, Suppl. Data S1). The 334 upregulated priming-responsive genes in *A. thaliana* play amongst others a role in phenylpropanoid biosynthesis and immune responses, including responses to SA (Fig. 5, Suppl. Data S2 and S3), which was shown to be crucial for establishing the egg-mediated anti-herbivore defense response in *A. thaliana* (Lortzing et al. 2019; Valsamakis et al. 2020).

*Arabidopsis lyrata* upregulated considerably fewer genes in response to eggs than *A. thaliana*. The rather moderate transcriptional response of *A. lyrata* to *P. brassicae* eggs might result in the absence of a priming response in *A. lyrata.* Although *A. lyrata* genes were responsive to eggs per se, this transcriptional change did not result in an additive or synergistic transcriptional interactions with the “larval feeding stimulus” (Fig. 6b). By contrast, *A. thaliana* responded much stronger to the *P. brassicae* eggs resulting in maintained egg-induced responses or additive and synergistic interactions between “egg stimulus” and “larval feeding stimulus” (Fig. 6b). Additive and synergistic effects contribute to the priming response of *A. thaliana* and result in an accelerated and stronger resistance response of *A. thaliana* against *P. brassicae* larvae if the plant is previously exposed to conspecific eggs (Lortzing et al. 2020; Valsamakis et al. 2020, 2022).

The expression of genes typically responsive to eggs, such as *PDF1.4, PR1*, and *PR5* (Little et al. 2007; Paniagua Voirol et al. 2020; Valsamakis et al. 2020) was significantly induced by eggs alone in both plant species (Fig. 4). By contrast, the expression of *CAX3* was strongly induced by eggs in *A. thaliana,* but not in *A. lyrata* (Fig. 4). *CAX3* encodes for a Ca^2+^/H^+^ exchanger localized in the tonoplast (Manohar et al. 2011). Thus, calcium signaling seems to play an important role in perceiving the *P. brassicae* eggs and establishing the egg-primed plant defense response against the larvae.

Furthermore, prior egg deposition on feeding-damaged *A. thaliana* resulted in upregulation of genes that are involved in oxidative stress (Suppl. Data S3). Calcium signaling and oxidative stress are linked (Ermak and Davies 2002). Oxidative stress occurs when the cellular redox status is disrupted, for example, due to the overproduction of reactive oxygen species (ROS), and causes Ca^2+^ influx into the cytoplasm (Ermak and Davies 2002). When applied onto plant wounds, insect-derived elicitors, e.g., inceptin and volicitin, induce a Ca^2+^ influx, which in turn triggers ROS production (Kumar et al. 2020). During stress such as herbivory, the level of cytosolic Ca^2+^ rapidly increases due to Ca^2+^ influx mediated by Ca^2+^-ATPases and Ca^2+^ efflux mediated by H^+^/ Ca^2+^ exchanger such as CAX1 and CAX3. CAX1 and CAX3 have high specificity for Ca^2+^ binding and maintenance of Ca^2+^ homeostasis (Kumar et al. 2020). Furthermore, ROS is known to locally accumulate at the oviposition site in various annual and perennial plant species (Little et al. 2007; Gouhier-Darimont et al. 2013; Reymond 2013; Bittner et al. 2017; Geuss et al. 2017). ROS accumulation and cytosolic Ca^2+^ increase might initiate *A. thaliana*’s response to eggs and contribute to mounting defenses against *P. brassicae* larvae. Besides, our results showed that both *A. thaliana* and *A. lyrata* upregulated their genes related to oxidative stress after egg deposition and feeding. However, *A. thaliana* responded broader and stronger, including a specific cellular stress response that *A. lyrata* did not show. This suggests that ROS is not accumulating in *A. lyrata* after egg deposition. Further investigation will shed light to the yet unknown role of CAXs and on how oxidative stress and calcium signaling are integrated to establish the egg-primed anti-herbivore defense in *A. thaliana.*

Although *A. lyrata* responds to *P. brassicae* eggs only with moderate transcriptional changes, its transcriptional response to larval feeding without prior egg deposition was robust (Fig. 2, Fig. 6a, Suppl. Data S1), which may indicate that *A. lyrata* favors rapid transcriptomic changes in response to larval feeding over priming. Whether the stronger transcriptomic response of *A.* *lyrata* to larval feeding compared to the response of *A. thaliana* is due to the larvae feeding more intensively on *A. lyrata* than on *A. thaliana* or whether this is due to a more sensitive response of *A. lyrata* to larval feeding requires further investigations. In response to larval feeding, both plant species upregulated genes that play a role in JA and SA synthesis and signaling as well as in the synthesis of flavonoids including anthocyanins. Furthermore, both plant species downregulated genes that play a role in photosynthesis (Fig. 5, Suppl. Data S2 and S3). Previous studies showed that plant defense responses to insect herbivore attack are associated with a reduction in photosynthesis, thus allowing plants to allocate resources toward immediate defense needs (Bilgin et al. 2010; Kerchev et al. 2012). However, regarding upregulated genes that are involved in SA-mediated signaling and downregulated genes that are involved in photosynthesis, *A. lyrata*’s response to larval feeding seems to be more pronounced than *A. thaliana*’s response. The stronger feeding-mediated induction of SA-responsive and defense-related genes was also reflected by the stronger expression of *PR1*, *PR5* and *PDF.1.4* in *A. lyrata* than in *A. thaliana* (Fig. 4). Additionally, *CAX3* expression was remarkably strong in response to larval feeding (Fig. 4). Therefore, Ca^2+^ signaling might be a key component for *A.* *lyrata’*s defense response against the *P. brassicae* larvae. A study from Toyota et al. (2018) revealed that feeding by *P. rapae* larvae induced cytosolic Ca^2+^ accumulation in *A. thaliana*. Thus, *P. brassicae* larval feeding may affect *A.* *lyrata* in a similar manner as *P. rapae* feeding on *A. thaliana*. Taken together, the massive transcriptome reprogramming of *A.* *lyrata* in response to larval feeding shows that *A. lyrata* has evolved a robust response against *P. brassicae* larval feeding that is independent of prior egg deposition.

Earlier studies have demonstrated that further perennial plant species can be primed by insect egg deposition (Beyaert et al. 2012; Pashalidou et al. 2015; Austel et al. 2016; Geuss et al. 2018). From a life history strategy perspective, priming their defenses at the time of herbivore oviposition would be advantageous for perennial plants. However, our findings indicate that this is not the case for *A. lyrata*. It is tempting to attribute the defense strategies of *A. thaliana* and *A. lyrata* to their respective natural habitats. Since priming against herbivory is costly (Valsamakis et al. 2022), growing in environments with low density of competing species (Mitchell-Olds 2001; Vergeer and Kunin 2011) may push *A. lyrata* to prioritize its stress responses, thus possibly saving resources for growth (Wise and Abrahamson 2007). If stress by herbivory occurs relative to other stress events less frequently in the habitat of *A. lyrata,* it might be cost-saving when limiting primability to the most frequent stress responses. If this is the case with *A. lyrata* habitats, it might explain why *A.* *lyrata* remains unaffected by *P.* *brassicae* eggs and exhibits a robust response once larvae start feeding. Conversely, given that *A. thaliana* prospers mostly in environments where nutrients and water are not limited (Al-Shehbaz and O’Kane 2002) and where herbivory might represent a more frequently occurring threat, for *A. thaliana* the more efficient strategy would be to invest in defense against herbivory after egg deposition, that is, by priming its defenses before the larvae hatch. Other egg-primable Brassicaceae species like *B. nigra* (Pashalidou et al. 2013), *B. oleracea* and *S.* *arvensis* (Pashalidou et al. 2015) also occur on soils where nutrient and water are usually not limited (Fogg 1950; Stace 1997).

In conclusion, our results suggest that the primability of anti-herbivore defenses against herbivory is not phylogenetically conserved in the genus *Arabidopsis*. In contrast to other perennial plants—including *B. oleracea* as a Brassicaceae species—*A. lyrata’s* anti-herbivore defense turned out to be not primable by insect egg deposition, indicating that the life span of a plant does not affect its egg primability (Beyaert et al. 2012; Pashalidou et al. 2015; Austel et al. 2016; Geuss et al. 2018). Future studies are needed to elucidate the traits that determine a plant’s sensitivity to insect eggs, which probably affect the plant’s egg primability. The balance between the frequency of insect infestations and other (a)biotic stresses in the natural habitats of plants might shape the selection for traits relevant for becoming primed by insect egg deposition. Among these traits, those responsive for perception of eggs and the specific role of *CAX3* in plant defense responses against herbivores needs to be addressed in future studies.

## Supplementary information

Below is the link to the electronic supplementary material.

Supplementary file1 (DOCX 398 KB)

Supplementary file2 (XLSX 4292 KB)

Supplementary file3 (XLSX 81 KB)

Supplementary file4 (XLSX 325 KB)

Supplementary file5 (XLSX 134 KB)

Supplementary file6 (XLSX 254 KB)

## Data Availability

All presented data are included in the article or are available as Supplementary data. The RNA-seq raw sequences data are deposited at the European Bioinformatics Institute (EBI) platforms ArrayExpress and Expression Atlas under the accession number E-MTAB-12653.

## Abbreviations

C: Control untreated plants
DEG: Differentially expressed genes
E: Egg-treated plants
F: Plants treated with larval feeding, without prior egg deposition
E + F: Plants treated with egg-deposition and subsequent larval feeding
GO: Gene ontology
JA: Jasmonic acid
KEGG: Kyoto Encyclopedia of Genes and Genomes
SA: Salicylic acid

## References

[CR1] Al-Shehbaz IA, O’Kane SL (2002) Taxonomy and phylogeny of Arabidopsis (Brassicaceae). Arabidopsis Book TAB1:e0001. 10.1199/tab.0001 10.1199/tab.0001 PMC3243115 22303187

[CR3] Appel HM, Cocroft RB (2014) Plants respond to leaf vibrations caused by insect herbivore chewing. Oecologia 175:1257–1266. 10.1007/s00442-014-2995-6 24985883 10.1007/s00442-014-2995-6 PMC4102826

[CR4] Austel N, Eilers EJ, Meiners T, Hilker M (2016) Elm leaves ‘warned’ by insect egg deposition reduce survival of hatching larvae by a shift in their quantitative leaf metabolite pattern. Plant Cell Environ 39:366–376. 10.1111/pce.12619 26296819 10.1111/pce.12619

[CR5] Bandoly M, Hilker M, Steppuhn A (2015) Oviposition by *Spodoptera exigua* on *Nicotiana attenuata* primes induced plant defence against larval herbivory. Plant J 83:661–672. 10.1111/tpj.12918 26096574 10.1111/tpj.12918

[CR6] Bandoly M, Grichnik R, Hilker M, Steppuhn A (2016) Priming of anti-herbivore defence in *Nicotiana attenuata* by insect oviposition: herbivore-specific effects. Plant Cell Environ 39:848–859. 10.1111/pce.12677 26566692 10.1111/pce.12677

[CR7] Beyaert I, Köpke D, Stiller J, Hammerbacher A, Yoneya K, Schmidt A, Gershenzon J, Hilker M (2012) Can insect egg deposition ‘warn’ a plant of future feeding damage by herbivorous larvae? Proc R Soc B 279:101–108. 10.1098/rspb.2011.0468 21561977 10.1098/rspb.2011.0468 PMC3223639

[CR8] Bilgin DD, Zavala JA, Zhu J, Clough SJ, Ort DR, DeLucia EH (2010) Biotic stress globally downregulates photosynthesis genes. Plant Cell Environ 33:1597–1613. 10.1111/j.1365-3040.2010.02167.x 20444224 10.1111/j.1365-3040.2010.02167.x

[CR9] Bittner N, Trauer-Kizilelma U, Hilker M (2017) Early plant defence against insect attack: involvement of reactive oxygen species in plant responses to insect egg deposition. Planta 245:993–1007. 10.1007/s00425-017-2654-3 28175992 10.1007/s00425-017-2654-3

[CR10] Bittner N, Hundacker J, Achotegui-Castells A, Anderbrant O, Hilker M (2019) Defense of Scots pine against sawfly eggs (*Diprion pini*) is primed by exposure to sawfly sex pheromones. Proc Natl Acad Sci USA 116:24668–24675. 10.1073/pnas.1910991116 31748269 10.1073/pnas.1910991116 PMC6900732

[CR11] Bolger AM, Lohse M, Usadel B (2014) Trimmomatic: a flexible trimmer for Illumina sequence data. Bioinformatics 30:2114–2120. 10.1093/bioinformatics/btu170 24695404 10.1093/bioinformatics/btu170 PMC4103590

[CR12] Bonnet C, Lassueur S, Ponzio C, Gols R, Dicke M, Reymond P (2017) Combined biotic stresses trigger similar transcriptomic responses but contrasting resistance against a chewing herbivore in *Brassica nigra*. BMC Plant Biol 17:127. 10.1186/s12870-017-1074-7 28716054 10.1186/s12870-017-1074-7 PMC5513356

[CR13] Bray NL, Pimentel H, Melsted P, Pachter L (2016) Near-optimal probabilistic RNA-seq quantification. Nat Biotechnol 34:525–527. 10.1038/nbt.3519 27043002 10.1038/nbt.3519

[CR14] Bruessow F, Gouhier-Darimont C, Buchala A, Metraux J-P, Reymond P (2010) Insect eggs suppress plant defence against chewing herbivores. Plant J 62:876–885. 10.1111/j.1365-313X.2010.04200.x 20230509 10.1111/j.1365-313X.2010.04200.x

[CR15] Cheng C-Y, Krishnakumar V, Chan AP, Thibaud-Nissen F, Schobel S, Town CD (2017) Araport11: a complete reannotation of the *Arabidopsis thaliana* reference genome. Plant J 89:789–804. 10.1111/tpj.13415 27862469 10.1111/tpj.13415

[CR16] Cipollini D, Purrington CB, Bergelson J (2003) Costs of induced responses in plants. Basic Appl Ecol 4:79–89. 10.1078/1439-1791-00134

[CR17] Clauss MJ, Koch MA (2006) Poorly known relatives of *Arabidopsis thaliana*. Trends Plant Sci 11:449–459. 10.1016/j.tplants.2006.07.005 16893672 10.1016/j.tplants.2006.07.005

[CR18] Conrath U, Beckers GJM, Flors V et al (2006) Priming: getting ready for battle. Mol Plant Microbe Interact 19:1062–1071. 10.1094/MPMI-19-1062 17022170 10.1094/MPMI-19-1062

[CR19] David WAL, Gardiner BOC (1962) Oviposition and the hatching of the eggs of *Pieris brassicae* (L.) in a laboratory culture. Bull Entomol Res 53:91–109. 10.1017/S0007485300047982

[CR20] Dicke M, Baldwin IT (2010) The evolutionary context for herbivore-induced plant volatiles: beyond the ‘cry for help.’ Trends Plant Sci 15:167–175. 10.1016/j.tplants.2009.12.002 20047849 10.1016/j.tplants.2009.12.002

[CR21] Ermak G, Davies KJA (2002) Calcium and oxidative stress: from cell signaling to cell death. Mol Immunol 38:713–721. 10.1016/S0161-5890(01)00108-0 11841831 10.1016/s0161-5890(01)00108-0

[CR22] Ewels P, Magnusson M, Lundin S, Käller M (2016) MultiQC: summarize analysis results for multiple tools and samples in a single report. Bioinformatics 32:3047–3048. 10.1093/bioinformatics/btw354 27312411 10.1093/bioinformatics/btw354 PMC5039924

[CR23] Fogg GE (1950) Sinapis arvensis L. J Ecol 38:415–429. 10.2307/2256459

[CR24] Fox J, Weisberg S (2019) An R companion to applied regression. Sage, Thousand Oaks CA

[CR25] Frost CJ, Mescher MC, Carlson JE, de Moraes CM (2008) Plant defense priming against herbivores: getting ready for a different battle. Plant Physiol 146:818–824. 10.1104/pp.107.113027 18316635 10.1104/pp.107.113027 PMC2259053

[CR26] Galili T (2015) dendextend: an R package for visualizing, adjusting and comparing trees of hierarchical clustering. Bioinformatics 31:3718–3720. 10.1093/bioinformatics/btv428 26209431 10.1093/bioinformatics/btv428 PMC4817050

[CR27] Garnier S, Ross N, Rudis B, Sciaini M, Camargo AP, Scherer C (2024) viridis(Lite)—Colorblind-friendly color maps for R. viridis package version 0.6.5. https://sjmgarnier.github.io/viridis/. 10.5281/zenodo.4679423

[CR28] Geiselhardt S, Yoneya K, Blenn B, Drechsler N, Gershenzon J, Kunze R, Hilker M (2013) Egg laying of cabbage white butterfly (*Pieris brassicae*) on *Arabidopsis thaliana* affects subsequent performance of the larvae. PLoS ONE 8:e59661. 10.1371/journal.pone.0059661 23527243 10.1371/journal.pone.0059661 PMC3602411

[CR29] Geuss D, Stelzer S, Lortzing T, Steppuhn A (2017) *Solanum dulcamara*’s response to eggs of an insect herbivore comprises ovicidal hydrogen peroxide production. Plant Cell Environ 40:2663–2677. 10.1111/pce.13015 28667817 10.1111/pce.13015

[CR30] Geuss D, Lortzing T, Schwachtje J, Kopka J, Steppuhn A (2018) Oviposition by *Spodoptera exigua* on *Solanum dulcamara* alters the plant’s response to herbivory and impairs larval performance. Int J Mol Sci 19:4008. 10.3390/ijms19124008 30545097 10.3390/ijms19124008 PMC6321313

[CR31] Gouhier-Darimont C, Schmiesing A, Bonnet C, Lassueur S, Reymond P (2013) Signalling of *Arabidopsis thaliana* response to *Pieris brassicae* eggs shares similarities with PAMP-triggered immunity. J Exp Bot 64:665–674. 10.1093/jxb/ers362 23264520 10.1093/jxb/ers362 PMC3542055

[CR32] Griese E, Caarls L, Bassetti N, Mohammadin S, Verbaarschot P, Bukovinszkine’Kiss G, Poelman EH, Gols R, Schranz ME, Fatouros NE (2021) Insect egg-killing: a new front on the evolutionary arms-race between brassicaceous plants and pierid butterflies. New Phytol 230:341–353. 10.1111/nph.17145 33305360 10.1111/nph.17145 PMC7986918

[CR33] Gu Z, Eils R, Schlesner M (2016) Complex heatmaps reveal patterns and correlations in multidimensional genomic data. Bioinformaics 32:2847–2849. 10.1093/bioinformatics/btw313 10.1093/bioinformatics/btw313 27207943

[CR34] Handley R, Ekbom B, Ågren J (2005) Variation in trichome density and resistance against a specialist insect herbivore in natural populations of *Arabidopsis thaliana*. Ecol Entomol 30:284–292. 10.1111/j.0307-6946.2005.00699.x

[CR35] Haukioja E, Suomela J, Neuvonen S (1985) Long-term inducible resistance in birch foliage: triggering cues and efficacy on a defoliator. Oecologia 65:363–369. 10.1007/BF00378910 28310440 10.1007/BF00378910

[CR90] Hayashi S, Ishii T, Matsunaga T, Tominaga R, Kuromori T, Wada T, Shinozaki K, Hirayama T (2008) The glycerophosphoryl diester phosphodiesterase-like proteins SHV3 and its homologs play important roles in cell wall organization. Plant Cell Physiol 49:1522–1535. 10.1093/pcp/pcn120 18718934 10.1093/pcp/pcn120

[CR36] Helms AM, de Moraes CM, Tröger A, Alborn HT, Francke W, Tooker JF, Mescher MC (2017) Identification of an insect-produced olfactory cue that primes plant defenses. Nat Commun 8:337. 10.1038/s41467-017-00335-8 28835618 10.1038/s41467-017-00335-8 PMC5569085

[CR37] Hilker M, Fatouros NE (2015) Plant responses to insect egg deposition. Annu Rev Entomol 60:493–515. 10.1146/annurev-ento-010814-020620 25341089 10.1146/annurev-ento-010814-020620

[CR38] Hilker M, Schwachtje J, Baier M et al (2016) Priming and memory of stress responses in organisms lacking a nervous system. Biol Rev 91:1118–1133. 10.1111/brv.12215 26289992 10.1111/brv.12215

[CR39] Hope RM (2022) Rmisc: ryan miscellaneous. R package version 1.5.1. https://CRAN.R-project.org/package=Rmisc. 10.32614/CRAN.package.Rmisc. Accessed 25 Apr 2024

[CR40] Howe KL, Contreras-Moreira B, de Silva N et al (2020) Ensembl Genomes 2020-enabling non-vertebrate genomic research. Nucleic Acids Res 48:D689–D695. 10.1093/nar/gkz890 31598706 10.1093/nar/gkz890 PMC6943047

[CR41] Hu TT, Pattyn P, Bakker EG et al (2011) The *Arabidopsis lyrata* genome sequence and the basis of rapid genome size change. Nat Genet 43:476–481. 10.1038/ng.807 21478890 10.1038/ng.807 PMC3083492

[CR42] Hwang S-Y, Liu C-H, Shen T-C (2008) Effects of plant nutrient availability and host plant species on the performance of two *Pieris* butterflies (Lepidoptera: Pieridae). Biochem Syst Ecol 36:505–513. 10.1016/j.bse.2008.03.001

[CR43] Kassambara A (2023) ggpubr: ‘ggplot2’ based publication ready plots. R package version 0.6.0. https://CRAN.R-project.org/package=ggpubr. 10.32614/CRAN.package.ggpubr. Accessed 25 Apr 2024

[CR44] Kerchev PI, Fenton B, Foyer CH, Hancock RD (2012) Plant responses to insect herbivory: interactions between photosynthesis, reactive oxygen species and hormonal signalling pathways. Plant Cell Environ 35:441–453. 10.1111/j.1365-3040.2011.02399.x 21752032 10.1111/j.1365-3040.2011.02399.x

[CR45] Kopylova E, Noé L, Touzet H (2012) SortMeRNA: fast and accurate filtering of ribosomal RNAs in metatranscriptomic data. Bioinformatics 28:3211–3217. 10.1093/bioinformatics/bts611 23071270 10.1093/bioinformatics/bts611

[CR46] Kost C, Heil M (2006) Herbivore-induced plant volatiles induce an indirect defence in neighbouring plants. J Ecol 94:619–628. 10.1111/j.1365-2745.2006.01120.x

[CR47] Kumar A, Panwar R, Singh A, Singh IK (2020) Role of calcium signalling during plant–herbivore interaction. In: Giri B, Sharma MP (eds) Plant stress biology: strategies and trends. Springer, Singapore, pp 491–510

[CR48] Larsson J (2024) eulerr: area-proportional euler and venn diagrams with ellipses. R package version 7.0.2. https://CRAN.R-project.org/package=eulerr. 10.32614/CRAN.package.eulerr. Accessed 25 Apr 2024

[CR49] Little D, Gouhier-Darimont C, Bruessow F, Reymond P (2007) Oviposition by pierid butterflies triggers defense responses in Arabidopsis. Plant Physiol 143:784–800. 10.1104/pp.106.090837 17142483 10.1104/pp.106.090837 PMC1803735

[CR50] Livak KJ, Schmittgen TD (2001) Analysis of relative gene expression data using real-time quantitative PCR and the 2(-Delta Delta C(T)) method. Methods 25:402–408. 10.1006/meth.2001.1262 11846609 10.1006/meth.2001.1262

[CR51] Lortzing V, Oberländer J, Lortzing T, Tohge T, Steppuhn A, Kunze R, Hilker M (2019) Insect egg deposition renders plant defence against hatching larvae more effective in a salicylic acid-dependent manner. Plant Cell Environ 42:1019–1032. 10.1111/pce.13447 30252928 10.1111/pce.13447

[CR52] Lortzing T, Kunze R, Steppuhn A, Hilker M, Lortzing V (2020) Arabidopsis, tobacco, nightshade and elm take insect eggs as herbivore alarm and show similar transcriptomic alarm responses. Sci Rep 10:16281. 10.1038/s41598-020-72955-y 33004864 10.1038/s41598-020-72955-y PMC7530724

[CR53] Lortzing V, Valsamakis G, Jantzen F, Hundacker J, Paniagua Voirol LR, Schumacher F, Kleuser B, Hilker M (2024) Plant defensive responses to insect eggs are inducible by general egg-associated elicitors. Sci Rep 14:1076. 10.1038/s41598-024-51565-y 38212511 10.1038/s41598-024-51565-y PMC10784483

[CR54] Louda S, Mole S (1991) Glucosinolates: Chemistry and ecology. In: Rosenthal GA, Berenbaum M (eds) Herbivores: Their interactions with secondary plant metabolites, 2nd edn. Academic Press, San Diego, pp 123–164

[CR55] Love MI, Soneson C, Hickey PF, Johnson LK, Pierce NT, Shepherd L, Morgan M, Patro R (2020) Tximeta: reference sequence checksums for provenance identification in RNA-seq. PLoS Comput Biol 16:e1007664. 10.1371/journal.pcbi.1007664 32097405 10.1371/journal.pcbi.1007664 PMC7059966

[CR56] Manohar M, Shigaki T, Mei H, Park S, Marshall J, Aguilar J, Hirschi KD (2011) Characterization of *Arabidopsis* Ca^2+^/H^+^ exchanger CAX3. Biochemistry 50:6189–6195. 10.1021/bi2003839 21657244 10.1021/bi2003839

[CR57] Mitchell-Olds T (2001) *Arabidopsis thaliana* and its wild relatives: a model system for ecology and evolution. Trends Ecol Evol 16:693–700. 10.1016/S0169-5347(01)02291-1

[CR58] Nasrallah ME (2000) *Arabidopsis* species hybrids - Emerging model systems for the analysis of species differences. J Plant Growth Regul 19:326–333. 10.1007/s003440000034

[CR59] Oñate-Sánchez L, Vicente-Carbajosa J (2008) DNA-free RNA isolation protocols for *Arabidopsis thaliana*, including seeds and siliques. BMC Res Notes 1:93. 10.1186/1756-0500-1-93 18937828 10.1186/1756-0500-1-93 PMC2613888

[CR60] Paniagua Voirol LR, Valsamakis G, Lortzing V, Weinhold A, Johnston PR, Fatouros NE, Kunze R, Hilker M (2020) Plant responses to insect eggs are not induced by egg-associated microbes, but by a secretion attached to the eggs. Plant Cell Environ 43:1815–1826. 10.1111/pce.13746 32096568 10.1111/pce.13746

[CR61] Pashalidou FG, Lucas-Barbosa D, van Loon JJA, Dicke M, Fatouros NE (2013) Phenotypic plasticity of plant response to herbivore eggs: effects on resistance to caterpillars and plant development. Ecology 94:702–713. 10.1890/12-1561.1 23687896 10.1890/12-1561.1

[CR62] Pashalidou FG, Fatouros NE, van Loon JJA, Dicke M, Gols R (2015) Plant-mediated effects of butterfly egg deposition on subsequent caterpillar and pupal development, across different species of wild Brassicaceae. Ecol Entomol 40:444–450. 10.1111/een.12208

[CR63] Pashalidou FG, Eyman L, Sims J, Buckley J, Fatouros NE, de Moraes CM, Mescher MC (2020) Plant volatiles induced by herbivore eggs prime defences and mediate shifts in the reproductive strategy of receiving plants. Ecol Lett 23:1097–1106. 10.1111/ele.13509 32314512 10.1111/ele.13509

[CR64] Pastor V, Luna E, Mauch-Mani B, Ton J, Flors V (2013) Primed plants do not forget. Environ Exp Bot 94:46–56. 10.1016/j.envexpbot.2012.02.013

[CR65] Peiffer M, Tooker JF, Luthe DS, Felton GW (2009) Plants on early alert: glandular trichomes as sensors for insect herbivores. New Phytol 184:644–656. 10.1111/j.1469-8137.2009.03002.x 19703113 10.1111/j.1469-8137.2009.03002.x

[CR66] R Core Team (2022) R: a language and environment for statistical computing. R Foundation for Statistical Computing, Vienna, Austria. https://www.R-project.org/. Accessed 25 Apr 2024

[CR67] Rasmann S, de Vos M, Casteel CL, Tian D, Halitschke R, Sun JY, Agrawal AA, Felton GW, Jander G (2012) Herbivory in the previous generation primes plants for enhanced insect resistance. Plant Physiol 158:854–863. 10.1104/pp.111.187831 22209873 10.1104/pp.111.187831 PMC3271773

[CR68] Rawat V, Abdelsamad A, Pietzenuk B, Seymour DK, Koenig D, Weigel D, Pecinka A, Schneeberger K (2015) Improving the annotation of *Arabidopsis lyrata* using RNA-Seq data. PLoS ONE 10:e0137391. 10.1371/journal.pone.0137391 26382944 10.1371/journal.pone.0137391 PMC4575116

[CR69] Revelle W (2024) psych: Procedures for psychological, psychometric, and personality research. R package version 2.4.3. Northwestern University, Evanston, Illinois, USA. https://CRAN.Rproject.org/package=psych. 10.32614/CRAN.package.psych. Accessed 25 Apr 2024

[CR70] Reymond P (2013) Perception, signaling and molecular basis of oviposition-mediated plant responses. Planta 238:247–258. 10.1007/s00425-013-1908-y 23748628 10.1007/s00425-013-1908-y PMC3722449

[CR71] Rondoni G, Bertoldi V, Malek R, Djelouah K, Moretti C, Buonaurio R, Conti E (2018) *Vicia faba* plants respond to oviposition by invasive *Halyomorpha halys* activating direct defences against offspring. J Pest Sci 91:671–679. 10.1007/s10340-018-0955-3

[CR72] Schott J, Jantzen F, Hilker M (2023) Elm tree defences against a specialist herbivore are moderately primed by an infestation in the previous season. Tree Physiol 43:1218–1232. 10.1093/treephys/tpad038 37010106 10.1093/treephys/tpad038 PMC10335851

[CR73] Sherman BT, Hao M, Qiu J, Jiao X, Baseler MW, Lane HC, Imamichi T, Chang W (2022) DAVID: a web server for functional enrichment analysis and functional annotation of gene lists (2021 update). Nucleic Acids Res 50:W216–W221. 10.1093/nar/gkac194 35325185 10.1093/nar/gkac194 PMC9252805

[CR74] Sletvold N, Ågren J (2012) Variation in tolerance to drought among Scandinavian populations of *Arabidopsis lyrata*. Evol Ecol 26:559–577. 10.1007/s10682-011-9502-x

[CR75] Sletvold N, Huttunen P, Handley R, Kärkkäinen K, Ågren J (2010) Cost of trichome production and resistance to a specialist insect herbivore in *Arabidopsis lyrata*. Evol Ecol 24:1307–1319. 10.1007/s10682-010-9381-6

[CR76] Soneson C, Love MI, Robinson MD (2015) Differential analyses for RNA-seq: transcript-level estimates improve gene-level inferences. F1000Res 4:1521. 10.12688/f1000research.7563.2 26925227 10.12688/f1000research.7563.1 PMC4712774

[CR77] Stace C (1997) New flora of the British Isles, 2nd edn. Cambridge University Press, Cambridge

[CR78] Stahl E, Brillatz T, Ferreira Queiroz E, Marcourt L, Schmiesing A, Hilfiker O, Riezman I, Riezman H, Wolfender J-L, Reymond P (2020) Phosphatidylcholines from *Pieris brassicae* eggs activate an immune response in Arabidopsis. Elife 9:e60293. 10.7554/eLife.60293 32985977 10.7554/eLife.60293 PMC7521926

[CR79] Toyota M, Spencer D, Sawai-Toyota S, Jiaqi W, Zhang T, Koo AJ, Howe GA, Gilroy S (2018) Glutamate triggers long-distance, calcium-based plant defense signaling. Science 361:1112–1115. 10.1126/science.aat7744 30213912 10.1126/science.aat7744

[CR80] Turner TL, Bourne EC, von Wettberg EJ, Hu TT, Nuzhdin SV (2010) Population resequencing reveals local adaptation of *Arabidopsis lyrata* to serpentine soils. Nat Genet 42:260–263. 10.1038/ng.515 20101244 10.1038/ng.515

[CR2] Wingett SW, Andrews S (2018) FastQ screen: a tool for multi-genome mapping and quality control. F1000Res 7:1338. 10.12688/f1000research.15931 30254741 10.12688/f1000research.15931.1 PMC6124377

[CR81] Valsamakis G, Bittner N, Fatouros NE, Kunze R, Hilker M, Lortzing V (2020) Priming by timing: *Arabidopsis thaliana a*djusts its priming response to Lepidoptera eggs to the time of larval hatching. Front Plant Sci 11:619589. 10.3389/fpls.2020.619589 33362842 10.3389/fpls.2020.619589 PMC7755604

[CR82] Valsamakis G, Bittner N, Kunze R, Hilker M, Lortzing V (2022) Priming of Arabidopsis resistance to herbivory by insect egg deposition depends on the plant’s developmental stage. J Exp Bot 73:4996–5015. 10.1093/jxb/erac199 35522985 10.1093/jxb/erac199 PMC9366327

[CR83] Vergeer P, Kunin WE (2011) Life history variation in *Arabidopsis lyrata* across its range: effects of climate, population size and herbivory. Oikos 120:979–990. 10.1111/j.1600-0706.2010.18944.x

[CR84] War AR, Paulraj MG, Ahmad T, Buhroo AA, Hussain B, Ignacimuthu S, Sharma HC (2012) Mechanisms of plant defense against insect herbivores. Plant Signal Behav 7:1306–1320. 10.4161/psb.21663 22895106 10.4161/psb.21663 PMC3493419

[CR85] Wickham H, Averick M, Bryan J et al (2019) Welcome to the tidyverse. J Open Source Softw 4:1686. 10.21105/joss.01686

[CR86] Wickham H (2007) Reshaping data with the reshape package. R package version 1.4.4. J Stat Softw 21:1–20. https://CRAN.R-project.org/package=reshape2. 10.32614/CRAN.package.reshape2. Accessed 25 Apr 2024

[CR87] Wickham H (2016) ggplot2: Elegant graphics for data analysis. Springer-Verlag, New York. https://ggplot2.tidyverse.org. 10.32614/CRAN.package.ggplot2. Accessed 25 Apr 2024

[CR88] Wilke CO (2022) cowplot: Streamlined plot theme and plot annotations for ‘ggplot2’. https://CRAN.R-project.org/package=cowplot. 10.32614/CRAN.package.cowplot. Accessed 25 Apr 2024

[CR89] Wise MJ, Abrahamson WG (2007) Effects of resource availability on tolerance of herbivory: a review and assessment of three opposing models. Am Nat 169:443–454. 10.1086/512044 17253430 10.1086/512044

